# Iron Insufficiency Compromises Motor Neurons and Their Mitochondrial Function in *Irp2*-Null Mice

**DOI:** 10.1371/journal.pone.0025404

**Published:** 2011-10-07

**Authors:** Suh Young Jeong, Daniel R. Crooks, Hayden Wilson-Ollivierre, Manik C. Ghosh, Rachid Sougrat, Jaekwon Lee, Sharon Cooperman, James B. Mitchell, Carole Beaumont, Tracey A. Rouault

**Affiliations:** 1 *Eunice Kennedy Shriver* National Institute of Child Health and Development, National Institutes of Health, Bethesda, Maryland, United States of America; 2 Nanobiology Lab, King Abdullah University of Science and Technology (KAUST), Thuwal, Saudi Arabia; 3 Department of Biochemistry, University of Nebraska, Lincoln, Nebraska, United States of America; 4 National Cancer Institute, National Institutes of Health, Bethesda, Maryland, United States of America; 5 INSERM U773, Centre de Recherche Biomédicale Bichat-Beaujon, Université Paris Diderot, Paris, France; University of Cincinnatti, United States of America

## Abstract

Genetic ablation of Iron Regulatory Protein 2 (*Irp2, Ireb2*), which post-transcriptionally regulates iron metabolism genes, causes a gait disorder in mice that progresses to hind-limb paralysis. Here we have demonstrated that misregulation of iron metabolism from loss of *Irp2* causes lower motor neuronal degeneration with significant spinal cord axonopathy. Mitochondria in the lumbar spinal cord showed significantly decreased Complex I and II activities, and abnormal morphology. Lower motor neurons appeared to be the most adversely affected neurons, and we show that functional iron starvation due to misregulation of iron import and storage proteins, including transferrin receptor 1 and ferritin, may have a causal role in disease. We demonstrated that two therapeutic approaches were beneficial for motor neuron survival. First, we activated a homologous protein, IRP1, by oral Tempol treatment and found that axons were partially spared from degeneration. Secondly, we genetically decreased expression of the iron storage protein, ferritin, to diminish functional iron starvation. These data suggest that functional iron deficiency may constitute a previously unrecognized molecular basis for degeneration of motor neurons in mice.

## Introduction

The central nervous system (CNS) is one of the highest-energy requiring areas of the body, consuming energy at a rate nearly seven times faster per tissue weight compared to non-CNS areas [1], and neurons are highly dependent on efficient mitochondrial ATP production [2]. The respiratory chain complexes of mitochondria depend on iron cofactors, as they include twelve iron-sulfur [Fe-S] cluster prosthetic groups, and several hemes [3]. Therefore, large amounts of iron are needed to fully constitute respiratory chain complexes in the CNS. However, excess iron can cause devastating toxic effects to cells through the Fenton reaction [4], and cellular iron homeostasis is accordingly highly regulated. In mammalian cells, two cytosolic proteins known as Iron Regulatory Proteins (IRPs) regulate intracellular iron homeostasis by binding to mRNA stem-loop sequences known as Iron Regulatory Elements (IREs) in target transcripts. These two IRPs, IRP1 and IRP2, bind to IREs and regulate expression of proteins involved in iron homeostasis including the iron importer, transferrin receptor 1 (TfR1), the iron storage protein ferritin, which is a heteropolymer composed of FtH and FtL monomers, an iron exporter, ferroportin, and other transcripts [5], [6].

Previously, we have reported that mice lacking *Irp2* (*Irp1^+/+^;Irp2^-/-^*) show neurodegenerative symptoms including hind-limb weakness, tremor, subtle kyphosis, and abnormal gait [7], [8]. Moreover, we also generated mice lacking one copy of *Irp1* and both copies of *Irp2* (*Irp1^+/-^;Irp2^-/-^*) and reported that there is an *Irp* gene dosage effect on severity of neurodegeneration, wherein onset of disease and severity of symptoms occurs earlier in *Irp1^+/-^;Irp2^-/-^* mice [9]. Both *Irp1^+/+^;Irp2^-/-^* and *Irp1^+/-^;Irp2^-/-^* mice show misregulation in proteins involved in iron metabolism, with neuronal loss in different brain areas including cerebellum and substantia nigra [7], [9], [10]. Interestingly, these mice also show microcytic anemia, which was attributed to low expression of TfR1 and concomitant low iron uptake in the erythroid precursor cells [11], [12].

Despite the multiple clinical and pathological problems previously observed in *Irp2*-null mice, we had not previously analyzed the integrity of upper and lower motor neurons and assessed their potential contributions to the loss of locomotion observed in the *Irp2*-null mice. In this study, we analyzed brain and spinal cord cells that are involved in the motor function in both *Irp1^+/+^;Irp2^-/-^* and *Irp1^+/-^;Irp2^-/-^* mice, and we found that lower motor neurons appear to be the cells that are most adversely affected in *Irp2*-null mice. Here, we performed multiple studies including immunostaining and spinal cord lysate analyses which suggested that loss of *Irps* may cause functional iron starvation in these cells and may thereby impair mitochondrial activity. Finally, we attempted to mitigate disease using two approaches, including chemical recruitment of IRP1 activity, or genetic reduction of ferritin H synthesis, and we observed that both interventions significantly decreased neuronal degeneration in *Irp2*-null mice.

## Results

### Lack of Iron regulatory proteins caused axonal degeneration in spinal cord

**Figure 1:**
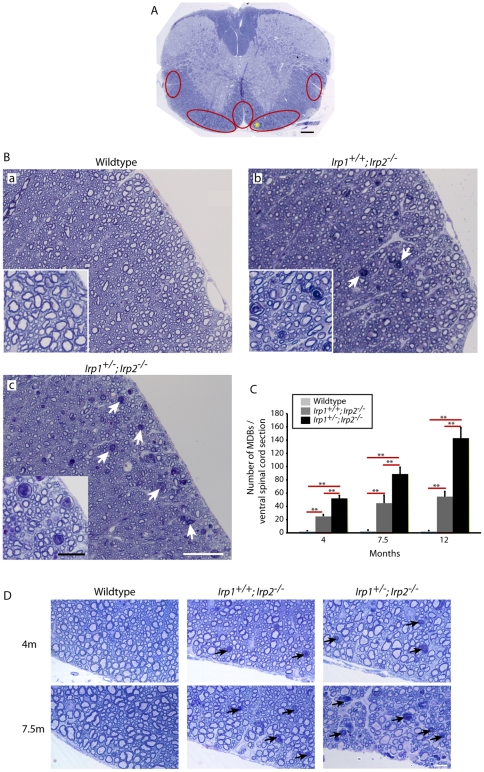
Axonal degeneration in the spinal cord of *Irp2*-null mice. A; Toluidine blue staining of Epon-embedded spinal cord cross-sections at 12 months. Red-circled areas indicate where myelin dense body (MDBs) were found. The yellow star represents the area enlarged in Figure 1B. Scale bar = 200 µm. B; Ventral white matter (L4) from wildtype (a), *Irp1^+/+^;Irp2^-/-^* (b), *Irp1^+/-^;Irp2^-/-^* (c) mice at 12 months. Arrows indicate MDBs. Scale bars = 50 µm, 20 µm (inset). C; Quantification of MDBs per spinal cord sections at 4, 7.5, 12 months. The bar chart show average±SEM, n = 4-5, **; p<0.001, analyzed by two-way ANOVA. A summary of the pairwise comparisons is presented in Table S1. D; Progressive accumulation of MDBs in ventral white matter of spinal cords at 4, 7.5 months. Scale bars = 10 µm.

As reported previously, *Irp2*-null mice showed abnormal neurological symptoms as they aged [7]. These symptoms include pronounced hind-limb weakness, poor weight bearing, tremor, and high muscle tone. To assess the integrity of neurons in the spinal cord from *Irp2*-null mice, we analyzed semi-thin plastic sections of lumbar spinal cord cross-sections (L4 level) from older adult (between 11 and 13 months of age) mice. We found massive accumulations of myelin dense bodies (MDB, also called myelin ovoids) in the ventral and lateral white matter of the *Irp1^+/-^;Irp2^-/-^* mice as well as in the *Irp1^+/+^;Irp2^-/-^* mice (Figure 1A, red circled areas, Figure 1Bb–c, arrows). MDBs are one of the hallmarks for neurodegeneration because they accumulate when axonal degeneration occurs and the myelin sheath collapses into the area formally occupied by the axon [13]. Figure 1B shows a higher magnification of the ventral white matter (asterisk area in Figure 1A). The number of MDBs significantly increased in the *Irp1^+/-^;Irp2^-/-^* mice compared not only to the wildtype, but also in comparison to the *Irp1^+/+^;Irp2^-/-^* mice (Figure 1C), which demonstrated a dose-dependent effect of *Irp-*null mutations. Moreover, the number of MDBs increased as mice aged. Mice of each genotype were analyzed at ages of 4, 7.5 and 12 months, and progressive degeneration of axons was quantified (Figure 1C-D, inter-age comparison is in Table S1). These data indicate that lack of *Irp2* causes axonal degeneration in mice, and severity of axonal degeneration increases in *Irp1^+/-^;Irp2^-/-^* mice.

### Degeneration of lower motor neurons

**Figure 2:**
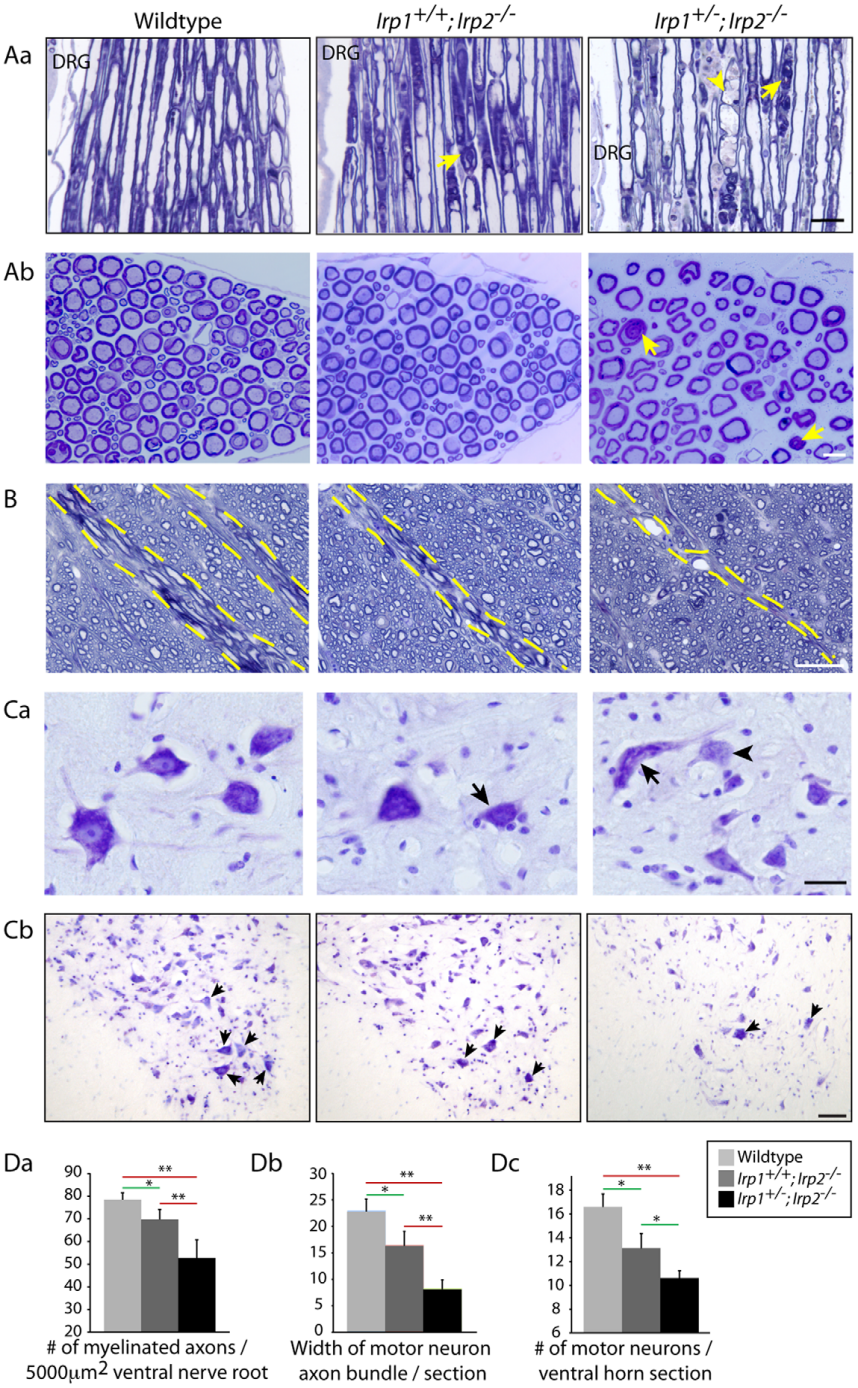
Atrophy and loss of lower motor neurons in the *Irp2-*null mice. Aa; Toluidine blue staining of Epon-embedded sections of ventral nerve root. Arrows show MDBs and arrowhead shows occasional swelling of axon. DRG; dorsal root ganglia. Scale bar = 25 µm. Ab; Cross section pictures of ventral nerve root. Arrows show MDBs. Scale bar = 10 µm. B; Motor neuronal axon exit area of the ventral white matter (dotted line). Scale bar = 30 µm. Ca; Cresyl violet staining showed distorted motor neuronal cell bodies (arrows) and motor neurons with chromatolysis (arrowhead) in the *Irp2*-null mice ventral horn. Scale bar = 25 µm. Cb; Lower magnification of ventral horn stained with cresyl violet. Arrows indicate motor neurons. Scale bar = 50 µm. Da; Quantification of number of myelinated fibers in the venral nerve root per 5000 µm^2^. n = 3,. Db; Quantification of motor neuronal axon bundles diameters per spinal cord. n = 5,. Dc; Quantification of motor neurons in the ventral horn stained with cresyl violet. n = 4, All data in D show average value±SEM per genotype, *; p<0.05, **; p<0.001, analyzed by one-way ANOVA.

The axons of lower motor neurons, which are involved in control of movements, cross through the ventral white matter to exit the spinal cord. Localization of the MDBs in the ventral and lateral spinal cord led us to hypothesize that some of the degenerating axons represented motor neuronal axons. To evaluate whether motor neuronal axons were affected, we analyzed ventral nerve roots located next to the dorsal root ganglion (DRG, Figure 2Aa), where these motor neuronal axons are known to exit the spinal cord. We found that there were increased numbers of swollen axons (Figure 2Aa, arrowhead) and also accumulations of MDBs (Figure 2A, arrows) in *Irp2*-null mice compared to wildtype. Moreover, the numbers of myelinated fibers in the ventral nerve roots were significantly decreased in *Irp2*-null mice (Figure 2Ab, Da, 78±3.1 myelinated fibers per 5000 µm^2^ for wildtype, 70±4.4 and 53±7.9 for *Irp2*-null mice). Then we analyzed the width of motor neuronal axon bundles in the ventral white matter. The diameter of the motor neuronal axon bundles was significantly decreased in *Irp2*-null mice (Figure 2B, Db, between dotted lines). Furthermore, when we analyzed the morphology of the motor neuronal cell bodies in the ventral horn by cresyl violet staining, *Irp2*-null mice showed several hallmarks of retrograde cell body degeneration, including distorted shape, rounding of cell bodies and loss of multipolarity, loss of Nissl body staining (chromatolysis, arrowhead), and eccentrically positioned nuclei (Figure 2Ca, arrows, [14]). There was also marked reduction in the number of large diameter cells observed when the ventral horn was stained with cresyl violet (Figure 2Cb). Finally, we quantified the number of motor neuronal cell bodies that stained with cresyl violet, and there was a significant decrease in the number of motor neuronal cell bodies in the *Irp2*-null mice compared to the wildtype (Figure 2Dc, 16.6±1.08 in wildtype, 13.1±1.23 in *Irp1^+/+^;Irp2^-/-^* and 10.6±0.64 in *Irp1^+/-^;Irp2^-/-^* per ventral horn section). We also analyzed the morphology of the dorsal nerve root where sensory axons are present, but there was no apparent pathology in the sensory axons (Figure S2A). These data demonstrate that motor neurons in the *Irp2*-null mice show significant degeneration, particularly if complete loss of *Irp2* is combined with heterozygous loss of *Irp1*, which corresponds well to the phenotype of these mice.

### Upper motor neuronal atrophy

**Figure 3:**
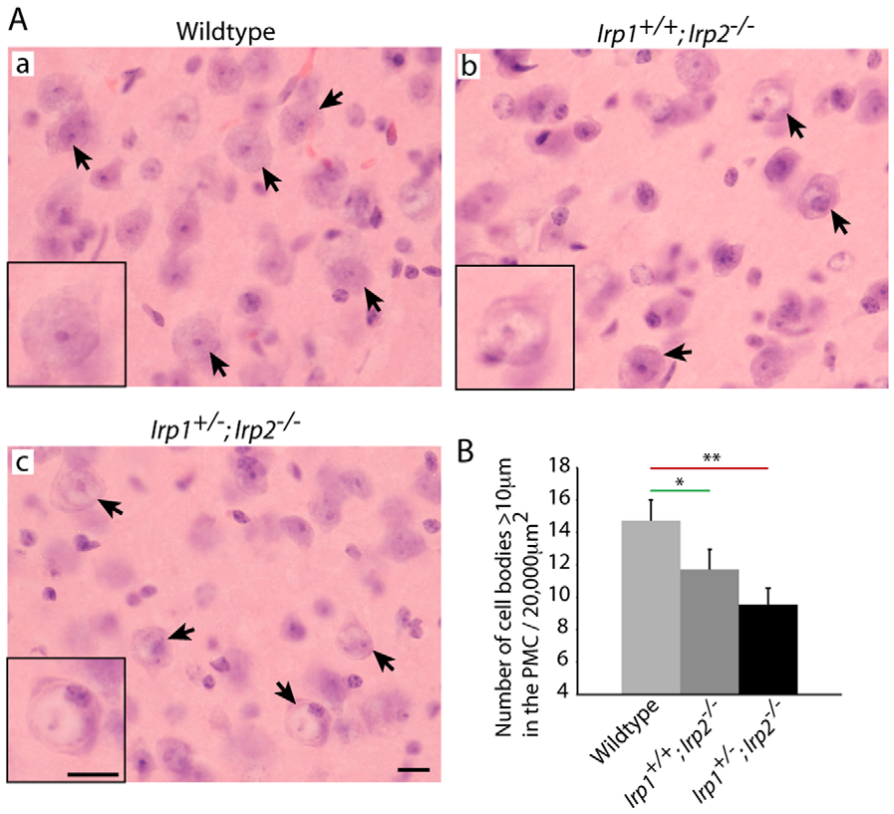
Status of primary motor cortex neurons in *Irp2*-null mice. A; H&E staining of the primary motor cortex (PMC). Arrows indicate large neurons in this area. Scale bar = 10 µm (both) B; Quantification of cell bodies that are larger than 10 µm in diameter / 20,000 µm^2^. n = 3. *; p<0.05, **; p<0.001, analyzed by one-way ANOVA.

Upper motor neurons connect between the motor cortex and spinal cord, and damage of upper motor neurons might contribute to the high muscle tone that we observed in *Irp2*-null mice. Accordingly, we analyzed morphology of cells in the primary motor cortex area by H&E staining and found fewer large diameter neurons (arrows) in *Irp2*-null mice (Figure 3A, B). In addition, H&E staining showed that there were more cells with chromatolysis (Figure 3A, inset), which is considered to be a marker for cell stress. Therefore, these data suggest that *Irp2*-null mice have not only lower motor neuronal degeneration, but also upper motor neuronal abnormalities.

### Stress markers are increased in the *Irp2*-null mice

**Figure 4:**
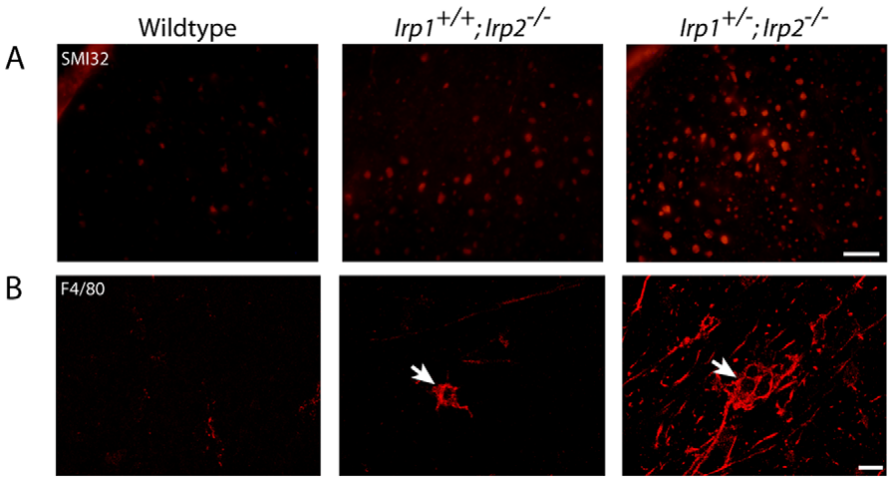
Increased expression of stress markers in the *Irp2*-null mice. A; Immunoreactivity for SMI32 (non-phosphoneurofilament antibody) was increased in *Irp2*-null mice. Scale bar = 30 µm. B; Infiltration of macrophages and/or microglia (F4/80 positive, arrows) was detected by confocal microscopy. Scale bar = 10 µm.

As shown in Figures 1 and 2, evidence for degeneration of lower motor neurons was observed in both axons and neuronal cell bodies in *Irp2*-null mice. We further evaluated motor neurons by examining expression of stress markers in both areas. First we stained sections using SMI32 (anti-non-phosphorylated neurofilament), which normally stains neuronal cell bodies but not healthy axons. As predicted, we could not detect immunoreactivity in axons of the wildtype, whereas a strong signal was detected in the lumbar ventral white matter of *Irp2*-null mice (Figure 4A). Also, there was a significant increase of anti-ubiquitin immunoreactivity in the motor neuronal cell bodies (Figure S3) of *Irp2*-null mice, suggesting that these neurons are under stress. Additionally, we found clusters of macrophages and/or microglia (positive for F4/80) by confocal microscopy in the *Irp1^+/-^;Irp2^-/-^* mice white matter, suggesting that immune cells were attracted into this area (Figure 4B, arrows). Thus, these data indicate that there is stress in the axons and motor neuronal cell bodies of the lumbar spinal cord of *Irp2*-null mice.

### Dysregulation of iron homeostasis proteins causes functional iron starvation

**Figure 5:**
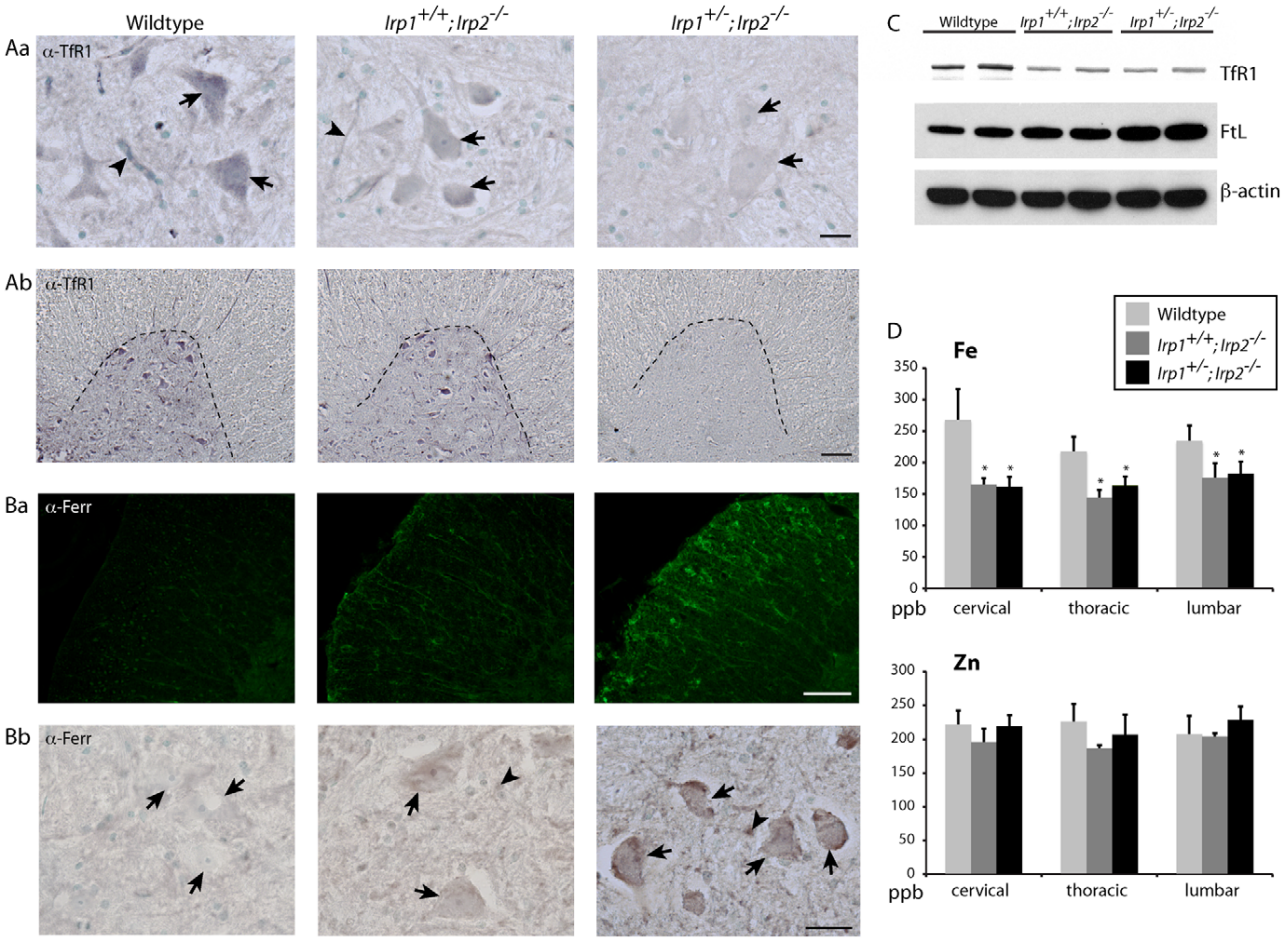
Abnormal expression of TfR1 and ferritin in motor neurons and glia of *Irp2*-null mice and diminished iron concentrations in spinal cord. Aa; Anti-TfR1 staining was decreased in *Irp2*-null mice motor neurons (arrows) and the endothelial cells of blood-brain barrier (arrowheads). Scale bar = 20 µm. Ab; Lower magnification picture of spinal cord ventral horn stained with anti-TfR1 antibody. Scale bar = 100 µm. B; Ferritin expression was prominent in both white (a) and grey matter (b) of the spinal cord. Motor neurons exhibited high ferritin expression (arrows) as did in glial cells (arrowheads). Scale bars = 100, 30 µm (respectively). C; Western blot analysis of TfR1 and Ferritin light chain from total spinal cord lysate. Beta-actin was used as a loading control. n = 5. D; Total tissue metal concentrations from different areas of spinal cord by ICP-MS reveal diminished Fe concentrations in the *Irp2*-null mice *; p<0.05, compared to wildtype, as analyzed by one-way ANOVA.

IRPs are key regulators of intracellular iron homeostasis. Under low iron conditions, these proteins bind to IREs to regulate expression of several transcripts that encode iron homeostasis proteins. When IRE-binding activity is diminished by genetic loss of *Irps*, expression of an iron importer, transferrin receptor 1 (TfR1), decreases and expression of the iron storage protein, ferritin, increases, and these changes can cause functional iron deficiency. Although the IRE/IRP regulatory system is ubiquitous in all cells, we found that the most significant misregulations of TfR1 and ferritin in the spinal cord were in the motor neurons. Anti-TfR1 staining showed significantly decreased TfR1 immunoreactivity (Figure 5Ab) in the motor neuronal cell bodies (Figure 5Aa, arrows) and endothelial cells of the blood-brain-barrier (BBB, Figure 5Aa, arrowheads, [1]) although all other cell types were also affected. Increased expression of ferritin was prominent in both white matter (Figure 5Ba), and in the motor neuronal cell bodies in the grey matter of spinal cord (Figure 5Bb, arrows). Some glial cells that likely represent inflammatory cells also showed increased expression of ferritin (Figure 5Bb, arrowheads). Changes in TfR1 and ferritin expression were also confirmed by Western blot analyses (Figure 5C). Finally we measured total tissue iron concentrations using ICP-MS and found that all three segments of spinal cord in *Irp2*-null mice had significantly lower amounts of iron than controls (Figure 5D) whereas there was no change in Zn levels. Therefore, we suggest that loss of *Irps* led to decreased iron uptake and increased iron sequestration in cells, which led to functional iron starvation in the spinal cords of *Irp2*-null mice. Moreover, the magnitude of misregulation appeared to be most significant in the motor neurons, which likely damaged these vulnerable cells.

### Mitochondrial dysfunction and atrophy caused by disrupted iron homeostasis

**Figure 6:**
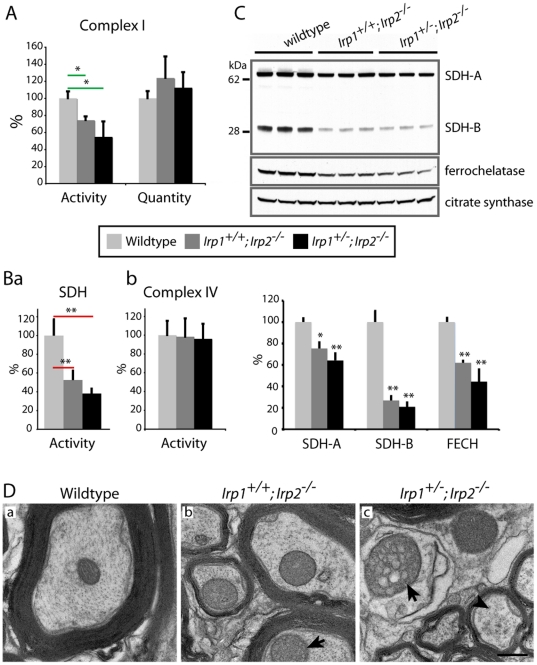
Mitochondrial dysfunction and atrophy in the *Irp2-*null mice. A; Complex I activity was decreased in *Irp2*-null mice whereas there were no changes in the amount of GRIM-19, a subunit of Complex I that was quantified. n = 6. Ba; SDH activity (Complex II) was markedly decreased in *Irp2*-null mice. Bb; There was no change in Complex IV activity which contains heme as a cofactor. *; p<0.05, **; p<0.001, analyzed by one-way ANOVA. C; The mitochondrial proteins SDH-A, SDH-B and ferrochelatase were significantly decreased in *Irp2*-null mice, whereas citrate synthase, which does not contain a [Fe-S] cluster, did not change and was used as a mitochondrial loading control. n = 5 **; p<0.001 compared to wildtype, analyzed by one-way ANOVA, D; TEM showed mitochondrial vacuolization (arrows) in axons in the ventral white matter of *Irp2*-null mice and abnormal bundling of neurofilaments within axons was also noted (arrowhead). Scale bar = 0.5 µm.

Iron is crucial for energy generation in mitochondria because respiratory chain complexes require [Fe-S] clusters and heme cofactors for function. Thus, functional iron starvation in *Irp2*-null mice might be expected to cause problems in mitochondrial respiratory chain activities. First, we assessed activity of respiratory chain Complex I, which contains eight [Fe-S] clusters. The activity of Complex I was significantly decreased in *Irp2*-null mice (Figure 6A, 74.4±4.19 and 54.7±18.03% compared to wildtype), whereas there were no changes in the amount of a key Complex I subunit protein, GRIM-19 (Figure 6A, [15]) as measured by a Complex I quantification kit. Moreover, activity of respiratory complex II, succinate dehydrogenase, which contains several [Fe-S] clusters in subunit B, was markedly decreased (SDH, Figure 6Ba), whereas there was no change in the activity of Complex IV, which does not contain [Fe-S] (Figure 6Bb). This effect of cellular iron starvation on [Fe-S] containing proteins was also confirmed by Western blot analyses, where we found marked reductions of the [Fe-S]-containing proteins SDH-B and ferrochelatase (FECH), and also mild reductions in SDH-A, which forms a complex with SDH-B, in the *Irp2-*null mice (Figure 6C). Decreased ferrochelatase has been observed previously in erythropoietic cells of *Irp2*-null mice [16], where decreased iron levels caused decreased stability of the protein. Consistent with these observations, total *Irp*-null mutations were recently reported to diminish respiratory chain complex activities in mouse livers [17].

In addition to abnormal mitochondrial respiratory chain activities, significant mitochondrial pathology was also observed in EM studies of *Irp2*-null mice. Abnormal mitochondria from *Irp2*-null mice were swollen, and had disrupted and vacuolized cristae (Figure 6D, arrows). Also, some axons showed evidence of mild demyelination (Figure 6Dc) and clustering of neurofilaments (Figure 6Dc, arrowhead), which are markers for neurodegeneration [18], [19]. Therefore, we concluded that functional iron starvation in *Irp2*-null mice neurons might be one of the causes of decreased mitochondrial function and atrophy.

### Therapeutic approaches; Tempol treatment

**Figure 7:**
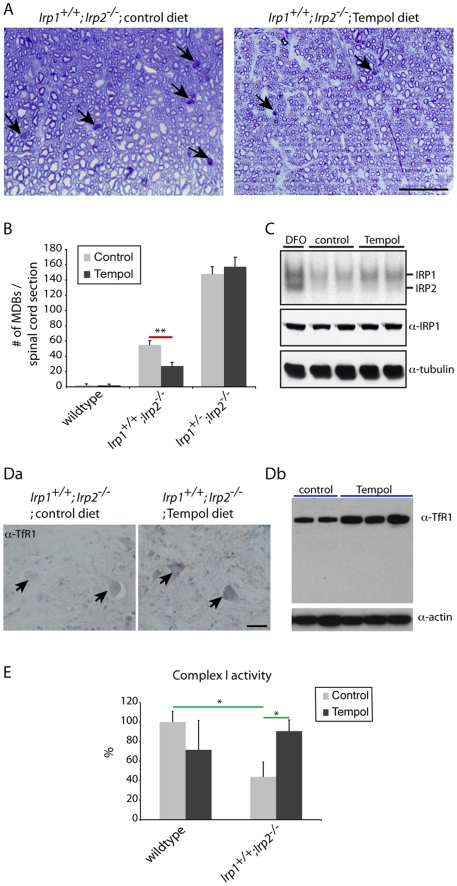
Dietary Tempol supplementation partially prevented axonal degeneration. A; Toluidine blue staining of Epon-embedded sections showed decreased MDBs (arrows) in the ventral white matter of Tempol treated mice. Scale bar = 50 µm. B; Quantification of MDBs per spinal cord section. n = 4, **; p<0.001, analyzed by two-way ANOVA. C; Gel-shift assay (top panel) show activation of IRP1 by Tempol treatment (100 µM) in wildtype mouse embryonic fibroblasts. The iron chelator, DFO, was used to show both IRP1 and IRP2 bands. Tempol treatment did not change total IRP1 (middle panel) by Western blot. Anti-tubulin antibody was used as a loading control. Da; Immunoreactivity for TfR1 was increased in the Tempol treated in the motor neurons of *Irp1^+/+^;Irp2^-/-^* mice (arrows). Db; Western blotting for TfR1 also showed increased expression of TfR1. E; Dietary Tempol treatment increased mitochondrial Complex I activity in *Irp1^+/+^; Irp2^-/-^* mice. n = 5, *; p<0.05, analyzed by two-way ANOVA.

As we reported previously, oral Tempol treatment attenuated neuromuscular compromise of the *Irp1^+/+^;Irp2^-/-^* mice [8]. Tempol is a stable nitroxide that readily crosses the blood brain barrier and it can act as an antioxidant, or as an iron-sulfur cluster destabilizing reagent [8], [20]. Here we further analyzed whether Tempol could prevent axonal degeneration in the spinal cord. We observed that the number of MDBs was significantly decreased in the Tempol-treated *Irp1^+/+^;Irp2^-/-^* mice (Figure 7A, B). This treatment also protected these mice from loss of neuromuscular skills as assessed by hang-tests ([8], Movie S1 or Figure S4). Interestingly, there was no significant beneficial effect of Tempol on *Irp1^+/-^;Irp2^-/-^* mice (Figure 7B), consistent with previous findings that Tempol could not prevent motor function decline in mice that had only one copy of *Irp1* [8]. In a previous paper, we have shown that Tempol destabilizes a synthetic [4Fe-4S] cluster *in vitro*, suggesting a mechanism for how Tempol activates IRP1 into the IRE-binding form. To confirm the effect of Tempol on activating IRP1, a gel-shift assay was performed using wildtype mouse fibroblasts. In this experiment, it was clear that Tempol converts IRP1 into the IRE-binding form without changing the total amount of IRP1 (Figure 7C). Likely as a consequence of this activation of IRE-binding activity, Tempol treatment increased expression of TfR1 in the motor neurons of *Irp1^+/+^;Irp2^-/-^* mice (Figure 7D), a change that might partially relieve these cells from functional iron starvation. Moreover, decreased mitochondrial Complex I activity in the *Irp1^+/+^;Irp2^-/-^* mice in the Tempol treated mice (Figure 7E) returned to near the levels of wildtypes. Therefore, these data suggest that motor neuronal degeneration might be caused by functional iron starvation, and that Tempol exerted its therapeutic effect in part by increasing expression of TfR1.

### Genetic modification to prevent motor neuron degeneration

**Figure 8:**
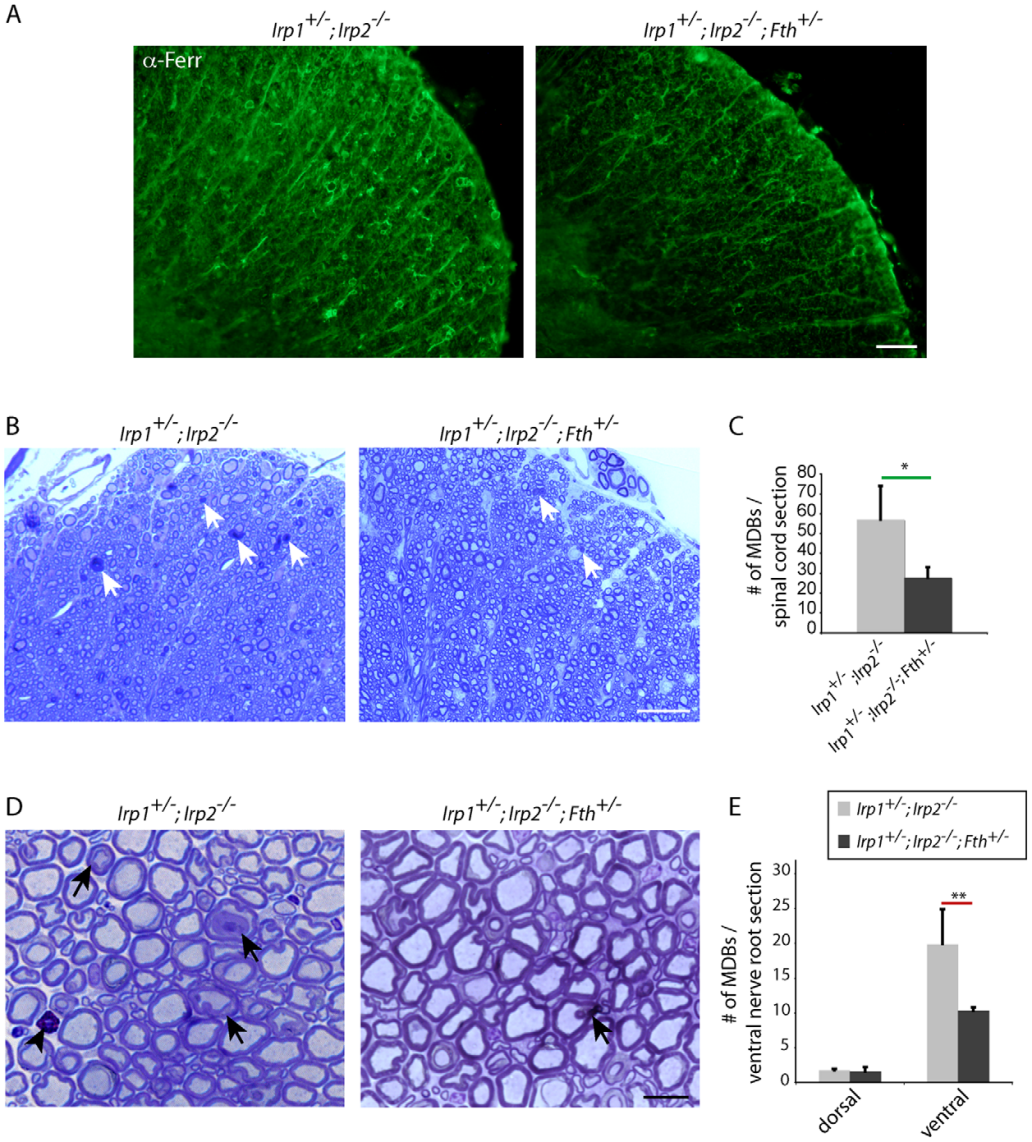
Decreased expression of ferritin H chain is beneficial for axonal survival. A; Anti-ferritin staining showed decreased expression of total ferritin in the spinal cord of *Irp1^+/-^;Irp2^-/-^;Fth^+/-^* mice. Scale bar = 50 µm. B; Toluidine blue staining of the ventral white matter showed decreased number of MDBs in the *Irp1^+/-^;Irp2^-/-^;Fth^+/-^ mice.* Scale bar = 30 µm. C; Quantification of MDBs per spinal cord section. The bar chart show average±SEM, n = 3, *; p<0.05, student's t-test. D; Toluidine blue staining of the cross section of ventral nerve root. *Irp1^+/-^;Irp2^-/-^;Fth^+/-^* mice showed decreased number of MDBs (arrows). Scale bar = 20 µm. E; Quantification of MDBs per nerve root section. The bar chart show average±SEM, n = 3, **; p<0.001, student's t-test.

As shown in Figure 5, loss of IRP activity not only decreased expression of TfR1 and limited iron uptake of cells, but also permitted increased expression of ferritin, which may have further contributed to functional iron starvation by sequestering iron within ferritin. Based on the abnormally high ferritin expression, we hypothesized that decreased expression of ferritin might be beneficial to these mice. To assess the role of ferritin, *Irp1^+/-^;Irp2^-/-^* mice were crossed with *Fth^+/-^* mice (Figure S1, [21]) to decrease expression of ferritin (Figure 8A), and MDBs in the ventral white matter were quantified. Perhaps due to the different genetic background of the *Fth^+/+^* mice, *Irp1^+/-^;Irp2^-/-^;Fth^+/+^* did not have as many MDBs as our *Irp1^+/-^;Irp2^-/-^* mice (Figure 8B,C, compared to Figure 1C); however *Irp1^+/-^;Irp2^-/-^;Fth^+/-^* mice showed approximately 50% sparing of axonal degeneration (57±16.9 vs. 27.7±5.24, Figure 8C) compared to their background-matched controls, demonstrating that decreased ferritin expression was beneficial in these mice. This sparing of axonal degeneration was prominent not only in the spinal cord but also in the ventral nerve root fibers, where MDBs were significantly decreased in the *Irp1^+/-^;Irp2^-/-^; Fth^+/-^* mice (Figure 8D, E). As mentioned above, we could not detect any significant axonopathies in the dorsal nerve roots (Figure 8E). Taken together, these data demonstrate that functional iron starvation due to abnormal iron homeostasis may be a major cause of motor neuronal degeneration in *Irp2*-null mice.

## Discussion

In this study, we report that disruption of iron homeostasis caused by *Irp2*-null mutations causes degeneration of motor neurons. One of the possible causes of this degeneration might be that functional iron starvation impairs the activity of mitochondrial respiratory chain complexes and disrupts mitochondrial integrity in these neurons. Moreover, two therapeutic approaches, including either oral Tempol treatment or genetic reduction of ferritin expression, delayed neurodegeneration, suggesting possible approaches to treatment if a human neurodegenerative disease attributable to loss of *IRP2* is identified in the future.

### Loss of Irps caused motor neuronal degeneration with mitochondrial atrophy

As we reported previously, *Irp2*-null mice showed neurodegenerative symptoms including tremor, hind-limb weakness, problems in weight bearing, and kyphosis [7], [9]. In these papers, we also reported abnormalities in the brain of *Irp2*-null mice including axonopathy and neuronal loss, but we did not analyze pathology in the spinal cord. Here we report that there is significant lower motor neuronal degeneration in the lumbar spinal cord of *Irp2*-null mice consistent with the marked gait abnormalities and hind-limb weakness (Figures 1, 2). This degeneration was more severe in the *Irp1^+/-^; Irp2^-/-^* mice than in the *Irp1^+/+^; Irp2^-/-^* mice, demonstrating a dose-dependent effect of *Irp*-null mutations. Moreover, analysis of motor cortex also suggested that there might be atrophy of upper motor neurons (Figure 3) that might contribute to the high-muscle tone that we observed in these mice, although we did not detect abnormalities in the dorsal corticospinal tract of the spinal cord (data not shown). However, we found accumulations of MDBs in the ventral funiculus where a minor portion of the corticospinal tract is proposed to run through the ventral white matter [22]. Moreover, the lateral localization of MDBs also suggested that neurons in the reticulospinal tract were adversely affected in the *Irp2*-null mice [22]. There was also minor demyelination of some axons and there were distorted mitochondria (Figure 6D), but we did not observe pathology in astrocytes or in oligodendrocytes (Figure S2B, C). Thus, it appears that motor neurons are the cells in the central nervous system that are most adversely affected by the disruption of iron homeostasis caused by loss of *Irp2*. Currently, it is not clear why motor neurons are more vulnerable than other neurons and glial cells to loss of *Irp2*. However, based on compromised respiratory complex activities in *Irp2*-null mice (Figure 6), it is possible that motor neurons depend more on IRP2 to maintain normal iron homeostasis and support mitochondrial function than other cells. Moreover, because motor neurons are the longest cells in the body, they are very dependent on mitochondrial activity to provide energy for ion pumps located along axons and concentrated at the nodes of Ranvier [23].

### Potential candidate gene for motor neuron diseases

To our knowledge, *IRP2* mutations have not yet been found in human patients with motor problems. Among motor neuron diseases, one example of disease that affects both upper and lower motor neurons is Amyotrophic Lateral Sclerosis (ALS). Recently, relationships between other iron homeostasis proteins and ALS have been reported in both mouse and human diseases [24], [25], [26], [27]. Therefore, it is interesting for us that null mutation in a key regulatory factor for iron homeostasis causes a phenotype comparable to human ALS and to mouse *SOD1* transgenic models. Our results show not only motor neuronal degeneration in the spinal cord, but also degeneration in the ventral root nerves (Figure 2), where motor neuronal axons run, as was reported in the *SOD1* mice [28]. Moreover, EM analysis showed significant vacuolization of mitochondria in axons of the *Irp2* null mice, similar to the *SOD1* mice [29]. It is also interesting that the large increase in serum ferritin levels observed in *Irp2*-null mice [30] was also observed in sporadic ALS patients [31]. However, even though *SOD1* mice and *Irp2*-null mice show somewhat similar phenotypes, iron metabolism may be misregulated in different ways, depending on the animal model. In some instances, misregulation can lead to cellular iron overload, as was reported for the spinal cords of *SOD1* mice [32] and treatment of the mice with an iron chelator was beneficial in these mice [32], [33]. However, our work here shows that *Irp2*-null mice seem to suffer from functional iron starvation, as evidenced by ICP-MS measurements of spinal cord lysates, caused by low TfR1 expression and high ferritin expression in motor neuron cell bodies. In conclusion, we suggest that *IRP2* might be a potential candidate gene for human diseases in which motor neuron problems predominate, based on our data that *Irp2*-null mice showed many pathologic traits that were reported in human patients, including axonal degeneration, accumulation of myelin dense bodies, lower and upper motor neuronal degeneration, and accumulation of ubiquitin positive aggregates in motor neurons.

### IRPs and potential relationship to human diseases

ALS is a complex disease with multiple possible genetic causes [34]. When Sreedharan *et al.* reported *TDP43* mutations in ALS patients, they also found an additional locus with LOD score (logarithm (base 10) of odds) that was higher than 1.0 at 15q23-q26 [32]. Interestingly, this locus is very close to *IRP2* (15q25), although the authors could not find contiguous markers nor could they identify a haplotype in this locus. Moreover, van Es *et al.* recently reported that chromosome 9p21.2 is a possible linkage locus for the sporadic form of ALS [35], and human *IRP1* is located at chromosome 9p21.1. Lastly, target gene searches for another movement disorder called Hereditary spastic paraplegia (HSP) resulted in identification of a disease gene at the 4p16-p15 locus (SPG 38, [36]), which is near a new E3 ubiquitin ligase called F-box and leucine-rich repeat protein 5 (FBXL5, 4p15.32). FBXL5 is a novel E3 ubiquitin ligase responsible for degradation of both IRPs [30], [37]. Therefore, if mutations in SPG38 changed stability of FBXL5, it might misregulate expression of both IRPs in cells. Taken together, these genetic data suggest that both *IRP*s and *FBXL5* are located near loci thought to contain human motor neuron disease genes.

### Rescue approaches

Previously, Tempol treatment has been shown to have beneficial effects on different mouse models [16], [38]. We also have shown that *Irp1^+/+^; Irp2^-/-^* mice treated with Tempol showed improvement in neuromuscular function and reduced iron burden in mouse cerebellum [8]. Here, we showed in further analysis that ventral white matter axons were partially spared from degeneration in the spinal cord of Tempol-treated *Irp2*-null animals (Figure 7A, B). As reported previously, Tempol treatment was ineffective for the *Irp1^+/-^; Irp2^-/-^* mice, suggesting that the salutary effect of Tempol might be due to activation of IRP1 ([8], Figure 7C), which led to increased expression of TfR1 (Figure 7D) and decreased expression of ferritin. Taken together, these data indicate that Tempol may be a good therapeutic reagent for animals with *Irp2*-null mutations.

As reported previously [7], *Irp2-*null mice show significant increase in iron storage protein ferritin expression. This upregulation was also noticed in the lumbar spinal cord, particularly in motor neurons (Figure 5). We also reported that oral treatments of Tempol decreased ferritin expression [8]. It has been reported that maintenance of proper levels of ferritin is important for reducing oxidative stress and enhancing neuronal survival [39], [40]. Therefore, we hypothesized that reduction of ferritin expression by eliminating one ferritin H allele might be beneficial for degenerating neurons. We demonstrated that *Irp1^+/-^; Irp2^-/-^* mice crossed with *Fth^+/-^* showed less neurodegeneration (Figure 8). Although there is no significant difference in total tissue iron between *Irp1^+/+^; Irp2^-/-^* and *Irp1^+/-^; Irp2^-/-^* mice (Figure 5D), we demonstrated that pathologic changes worsen in *Irp1^+/-^; Irp2^-/-^* mice. These data suggest that one of the reasons that motor neurons die in the *Irp2-*null mice is that the size of their ‘functional’ iron pool (compared to ‘total’) is reduced due to diminished TfR1 expression and increased ferritin expression. Reduced expression of TfR1 decreases iron uptake into cells, and increased expression of ferritin leads to increased sequestration of iron within ferritin heteropolymers. This functional iron deprivation likely impairs mitochondrial viability and function, and causes motor neuronal dysfunction and loss in these mice.

## Materials and Methods

### Animals and genotyping

Mice lacking *Irp*(s) were generated by targeting *AcoI* (*Irp1*) and *Ireb2* (*Irp2*) genes [7], [9], [41]. Genotypes of mice were determined by Southern blotting using gene-specific probes (Figure S1B). These mice have mixed genetic backgrounds consisting of C57BL/6 and B129S4/SVJ. Mice were anesthetized between 11-13 months of age and all the experimental protocols used in this study followed NICHD ACUC (*Eunice Kennedy Shriver* National Institute of Child Health and Human Development Animal Care and Use Committee) guideline and approved by the same committee (protocol number 09-038). For the Tempol experiments, mice were fed with either control or Tempol containing diet (10 mg/g, [8]) from the time of weaning until the time of sacrifice. Mice lacking one copy of ferritin H chain (*Fth^+/-^*, [21]) were provided by Dr. Carole Beaumont (INSERM U773, France). For the genotyping of *Fth^+/-^* mice, tissue was collected by ear clipping and incubated in an X-gal containing buffer for 2 hours at 37°C. When the beta-galactosidase gene was expressed under control of the *Fth* promoter, blue color was generated (Figure S1C) in *Fth^+/-^* mice. *Fth^+/-^* mice were crossed to *Irp*-null mice to generate *Irp1^+/-^;Irp2^-/-^;Fth^+/-^* mice (Detailed mating strategy is described in Figure S1A). *Fth* mice have C57BL/6 and 129SV mixed background. Background and age-matched control mice were used for comparison (2–11 days difference in age between genotypes). For each experiment, ‘n’ indicates number of animals used per genotype.

### Histology

Mice were deeply anesthetized and perfused with 2.5% glutaraldehyde (EM grade, Electron Microscopic Sciences) in 0.1 M sodium cacodylate buffer, pH 7.4. Dissected tissue samples were post-fixed and treated with 1.3% osmium tetroxide solution with 0.1 N potassium ferrocyanide. Samples were embedded in Epoxy Resin (EMS). Semithin (0.7 µm) sections were obtained using an ultramicrotome (Leica Microsystems) and stained with Epoxy Tissue Stain solution (EMS) for structural analysis. Some of the *Irp*-null mice spinal cord and longitudinal-cut root nerve sections were processed by the Laboratory for Neurotoxicity Studies at Virginia Tech (Blacksburg, VA, [7]). Spinal cord and root nerve sections were examined using bright field microscopy (Nikon Instruments). The number of myelin dense bodies was counted in the ventral and lateral white matter (n = 4−5 per genotype).For the motor cortex analysis, 35 µm sections were stained with Hematoxylin and Eosin by Dr. Robert Switzer at Neuroscience, Ltd. (Knoxville, TN, [9]). Neuronal cell bodies that were larger than 10 µm in diameter were quantified in the primary motor cortex. For the cresyl violet staining, 14 µm frozen sections from mouse spinal cord and brain were incubated with 0.1% cresyl violet solution for 10 min. For demyelination analysis, spinal cord sections were first dehydrated and immersed in 0.1% Luxol Fast Blue solution overnight at 37°C followed by chilling at 4°C for 30 min on the next day. After washing, slides were incubated in 0.05% lithium carbonate solution for 5 min, dehydrated and then mounted.

### Immunohistochemistry

*Irp2-*null and control mice were anesthetized as above and perfused with PBS and 4% paraformaldehyde, and 14 µm cryostat sections were then obtained. Immunolabeling of tissue sections was performed as described previously [32]. Briefly, tissue sections were incubated with PBS containing 2% normal goat serum and 1% ovalbumin to block nonspecific binding of antibodies. This was followed by an overnight incubation with mouse anti-TfR1 (1∶200, Invitrogen) or rabbit anti-ferritin antibody (1∶1000; gift from Dr. Esther Meyron-Holtz, Israel Institute of Technology, Haifa, Israel) or a rabbit anti-ubiquitin (1∶200; Dako). After washing, primary antibodies were recognized using biotin-conjugated secondary antibodies and signal was amplified using Vectastain ABC kit (Vector Lab). Immunoreactivity was visualized using 0.5 mg/ml 3,3′- diaminobenzidine as a chromagen (Sigma). Methyl green counterstain (20 mg/ml, Acro Chemicals) was used to visualize nuclei.

### Immunofluorescence

Spinal cord sections of mice were prepared as above and incubated with rat F4/80 (1∶100, Serotec), SMI32 (anti-non-phosphorylated neurofilament, 1∶500, Covance), anti-ferritin, or anti-GFAP (1∶100, Dako) overnight. After washing, sections were incubated with Alexa Fluor-conjugated secondary antibodies (1∶400, Invitrogen). 4′-6-diamidino-2-phenylindole (DAPI, 100 ng/ml, Vector Lab) was used to counterstain nuclei.

### Western blotting

Western blots were performed using total spinal cord lysates. Anti-TfR1 (1∶1000, Zymed), rabbit anti-FtL, anti-SDH-A, anti-SDH-B (1∶2000, Mitosciences), anti-ferrochelatase (1∶5000, [16]), anti-IRP1 (1∶5000, [8]) were used to detect antigen. Anti-actin (1∶400, Sigma), anti-tubulin (1∶5000, Sigma) and anti-citrate synthase (1∶10,000, Sigma) antibodies were used to confirm equal loading.

### Measuring tissue metals

Animals were deeply anesthetized and blood was removed by extensively perfusing PBS through the heart. Spinal cord samples were dissected in three segments (cervical, thoracic, lumbar) and snap frozen in liquid nitrogen. Total iron and zinc concentrations were measured by inductively coupled plasma mass spectroscopy (ICP-MS) as described previously [42] and normalized by wet tissue weight (n = 3).

### Mitochondrial respiratory complex assays

Activities and/or quantities of Complexes I and IV from the mitochondrial respiratory chain were assessed using specific Dipstick assay kits (MitoSciences) following the manufacturer's protocol. Briefly, snap-frozen tissue samples were homogenized and protein was extracted. After protein assay, 2 ug of total protein was used for the activity assay of Complex I (12 ug for the Complex IV). 20 ug of total protein was used to detect the level of GRIM-19 in Complex I (Complex I quantity assay kit, Mitosciences). Average readings of band densities were obtained using ImageJ (http://rsb.info.nih.gov/ij/) and data was plotted against wildtype value as 100%. n = 6 per genotype, two separate measurements per animal. Succinate dehydrogenase (SDH) activity was measured according to a previously published method [19] with modifications. Briefly, 50 ug of total spinal cord protein was mixed with a reaction buffer containing 50 mM Tris (pH 8.0), 0.5 mM ethylenediaminetetraacetic acid, 12 g/L Cremaphor EL (Sigma), 2 mM iodonitrotetrazolium chloride, 20 mM sodium succinate, 2 mM potassium cyanide, and 1 mM sodium azide. The reaction was followed at 492 nm for 10 min using a spectrophotometer (Thermo) at room temperature. Blank reactions without succinate were included to assess background activity. Assay specificity was verified by pre-incubation of some samples with the SDH inhibitor 3-nitropropionic acid (10 mM) for 30 minutes, which resulted in negligible residual enzymatic activity (data not shown). Data are presented as first-order kinetic rates normalized to the average of the wildtype sample values, as percent of control. Statistical analysis was performed on the raw, non-normalized data. n = 6 per genotype.

### Electron microscopy

Ultrastructural analysis of mitochondria in axons was performed in the ventral white matter following previously reported protocols [34]. Briefly, Epon-embedded spinal cord blocks were generated as described above and plastic sections were generated using an ultramicrotome (Leica). Sections were stained using lead citrate and analyzed by a Transmission Electron Microscope (Tecnai T20, FEI Company).

### Gel-shift assay

To test the effect of Tempol on IRP activation, wildtype mouse embryonic fibroblasts were treated with control media, control media plus Tempol (100 mM), and iron chelator DFO (100 mM) for 15 hours. Cells were collected and total protein was extracted using an NP-40 (0.2%) containing buffer. Gel-shift assays were performed using a ^32^P-labeled IRE probe following a protocol published previously [3].

### Mouse hang-test

The hang-test for assessment of motor function was performed in a blinded manner as published previously [8]. Briefly, each mouse was put on a wire mesh, which was then gently inverted. A video camera was used to record how long the mouse was able to hang on to the wire by clutching with the upper and lower extremities.

### Statistical analysis

Statistical significance was determined using a one-way or two-way ANOVA, as appropriate, and Tukey's post-hoc test was applied (SigmaPlot 12, Systat Software Inc). Two-tailed student's t-tests were applied for the pairwise comparisons in Figures 8C and 8E. (*; p<0.05, **; p<0.001).

## Supporting Information

Figure S1 **Generation of** ***Irp1^+/-^;Irp2^-/-^;Fth^+/-^*** **mice.** A; A schematic diagram showing four generations of mating strategy to generate *Irp1^+/-^;Irp2^-/-^;Fth^+/-^* and control mice (red box). Asterisks indicate embryonically lethal genotypes. P; parents, F1-3; progeny generation 1–3. B; Genotyping analysis by Southern blot showed specific bands for *Irp1* and *Irp2* (10.1 and 15.1 kb, respectively). Upon targeted deletion, each probe detected a shorter band (4.1 and 3.5 kb, respectively). C; Beta-galactosidase reporter assay distinguished *Fth^+/-^* (blue color, bottom) from wildtype (top).
(PDF)

Figure S2 **No significant pathological changes were observed in dorsal root nerve fibers and glia of** ***Irp2*** **-null mice.** A; Toluidine blue staining of Epon-embedded sections from mouse dorsal root nerve do not show significant degeneration in this area. B; Luxol Fast Blue staining of mouse ventral white matter does not show significant demyelination. C; anti-GFAP staining was performed to examine reactive astrocytes in ventral white matter. Immunoreactivity was not significantly increased in *Irp2-*null mice. Scale bars = 5 µm, 50 µm, 100 µm.
(PDF)

Figure S3 **Motor neurons (arrows) in ventral horn of** ***Irp2*** **-null mice showed increased ubiquitin expression.**
(PDF)

Figure S4 **Screen capture from Movie S1 showing beneficial effect of Tempol on mice neuromuscular behavior.**
(PDF)

Movie S1 **Beneficial effect of Tempol in** ***Irp1^+/+^;Irp2^-/-^*** **mice.** Mice treated with Tempol diet (right) since weaning showed spared motor skill compared to the control diet mice (left). *If the supplemental movie does not play, please see the screen captures in Figure S4 or go to http://science.nichd.nih.gov/confluence/download/attachments/44106370/13controlandTempolcombinedsmall.mov?version=1&modificationDate=1285279775000.
(MOV)

Table S1 **Summary of two-way ANOVA pairwise multiple comparisons (Tukey's Test), computed to evaluate the effect of age and genotype on the number of Myelin Dense Bodies (MDBs) in ventral spinal cord sections (average values are summarized in** **Figure 1C** **).** Two factors (age, genotype) were used for comparison and p<0.05 was used for statistical significance.
(PDF)
