# Supplementary figures and images for: Iron Insufficiency Compromises Motor Neurons and Their Mitochondrial Function in Irp2-Null Mice
Source: PLoS One. 2011 Oct 7;6(10):e25404. doi: 10.1371/journal.pone.0025404 (PMC3189198; doi:10.1371/journal.pone.0025404)

Figure S1

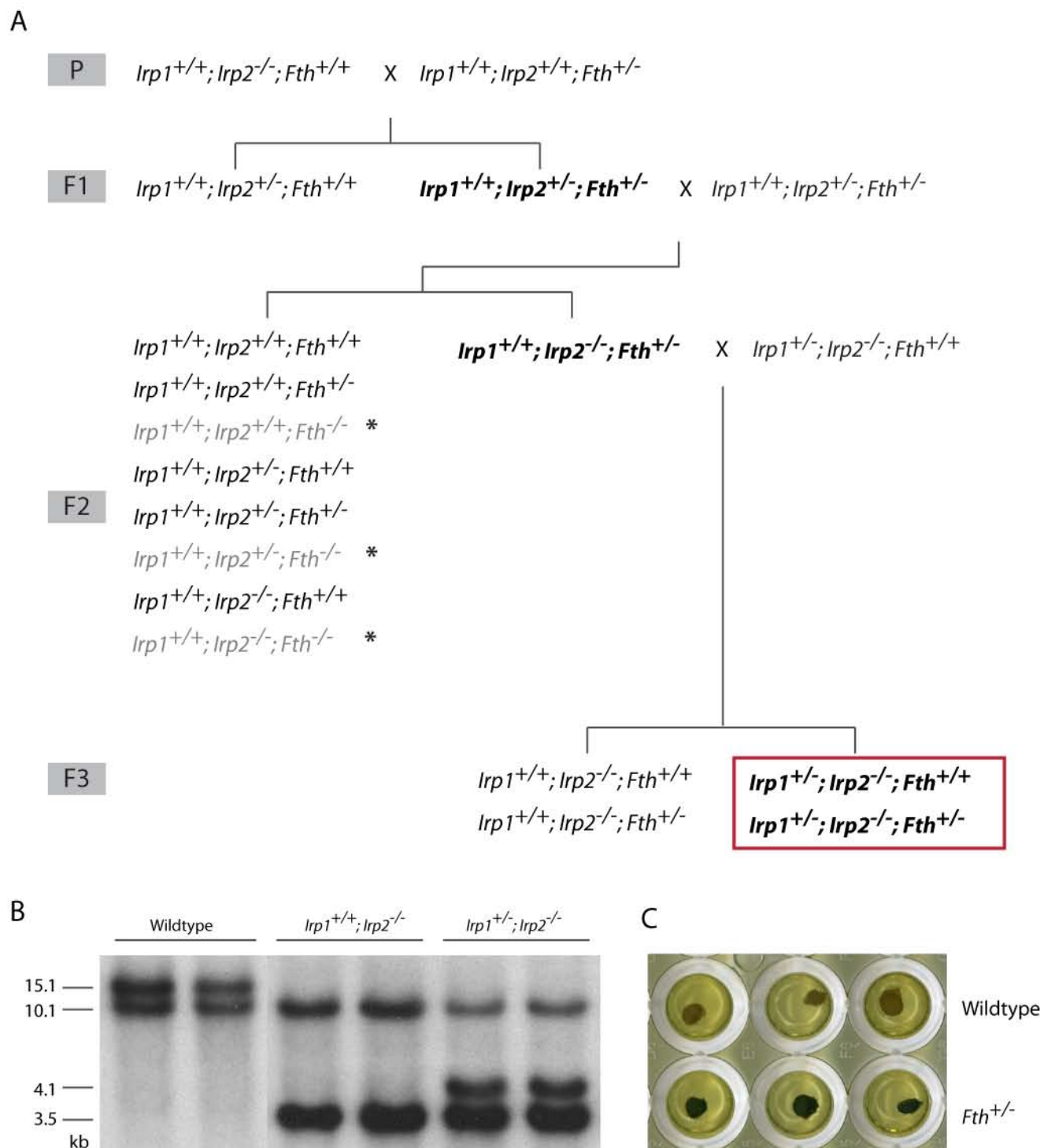

Supplement: Figure S1 — Generation of Irp1+/-;Irp2-/-;Fth+/- mice. A; A schematic diagram showing four generations of mating strategy to generate Irp1+/-;Irp2-/-;Fth+/- and control mice (red box). Asterisks indicate embryonically lethal genotypes. P; parents, F1-3; progeny generation 1–3. B; Genotyping analysis by Southern blot showed specific bands for Irp1 and Irp2 (10.1 and 15.1 kb, respectively). Upon targeted deletion, each probe detected a shorter band (4.1 and 3.5 kb, respectively). C; Beta-galactosidase reporter assay distinguished Fth+/- (blue color, bottom) from wildtype (top). (PDF) [file pone.0025404.s001.pdf]

Figure S2

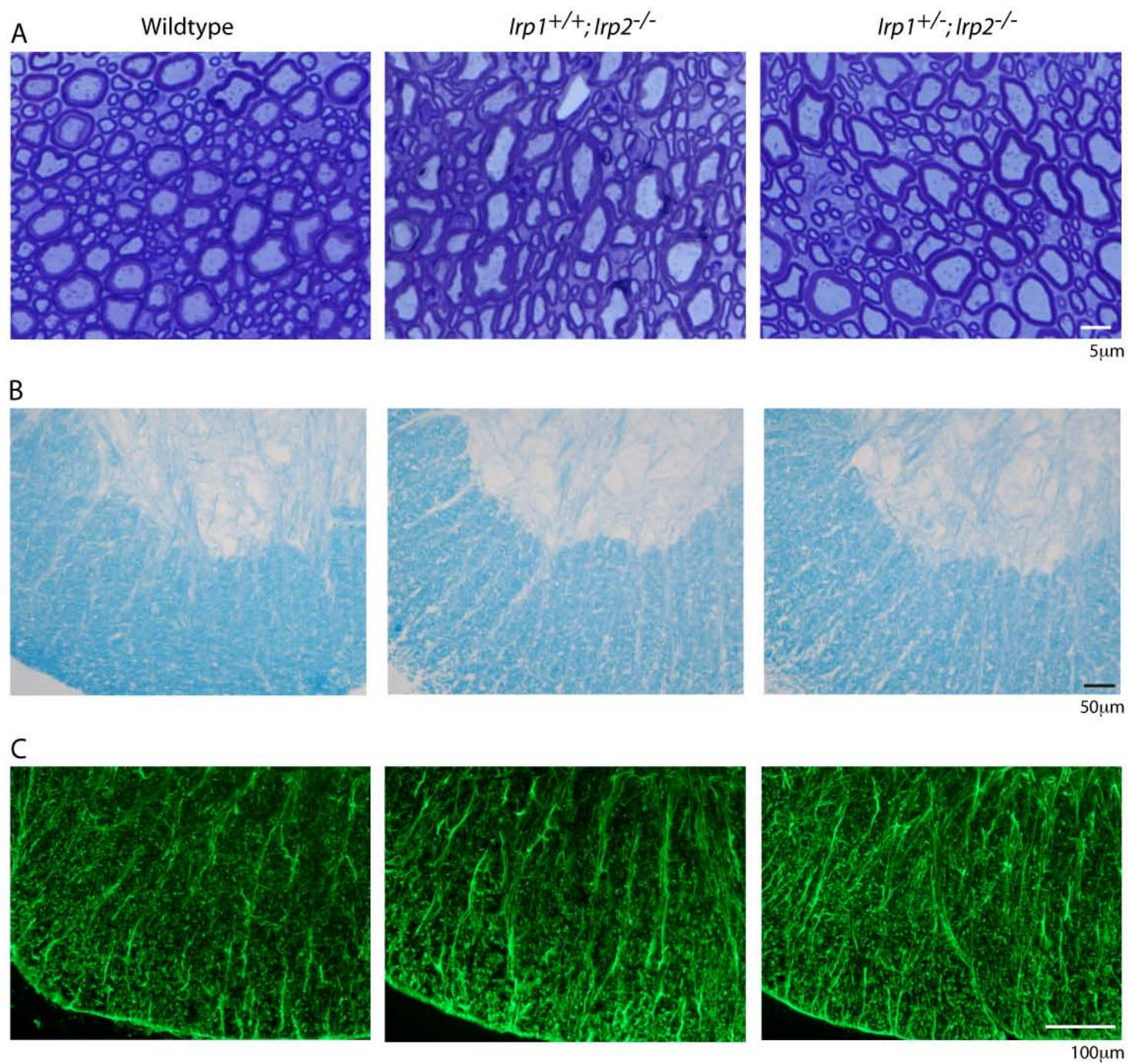

Supplement: Figure S2 — No significant pathological changes were observed in dorsal root nerve fibers and glia of Irp2 -null mice. A; Toluidine blue staining of Epon-embedded sections from mouse dorsal root nerve do not show significant degeneration in this area. B; Luxol Fast Blue staining of mouse ventral white matter does not show significant demyelination. C; anti-GFAP staining was performed to examine reactive astrocytes in ventral white matter. Immunoreactivity was not significantly increased in Irp2-null mice. Scale bars = 5 µm, 50 µm, 100 µm. (PDF) [file pone.0025404.s002.pdf]

Figure S3

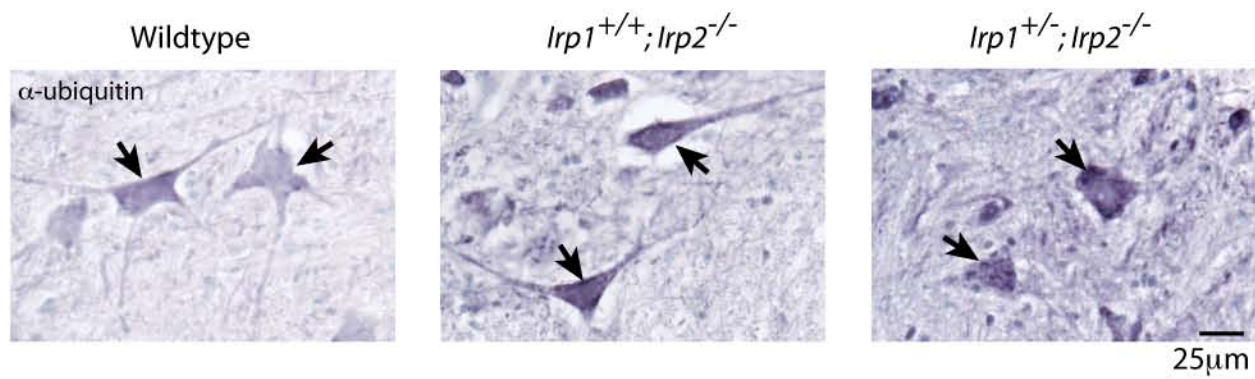

Supplement: Figure S3 — Motor neurons (arrows) in ventral horn of Irp2 -null mice showed increased ubiquitin expression. (PDF) [file pone.0025404.s003.pdf]

Figure S4

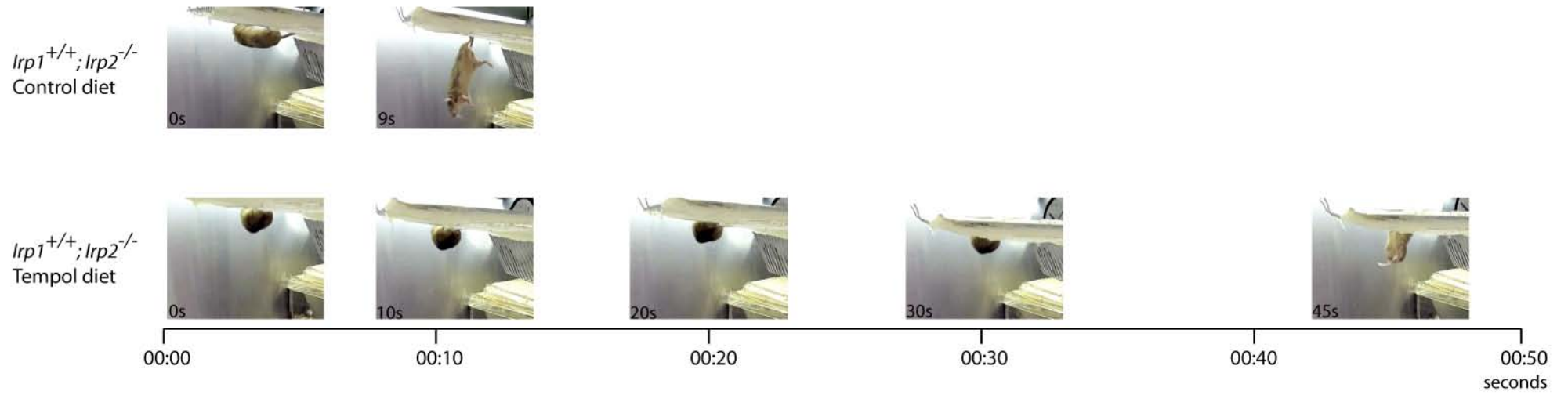

Supplement: Figure S4 — Screen capture from Movie S1 showing beneficial effect of Tempol on mice neuromuscular behavior. (PDF) [file pone.0025404.s004.pdf]
