# Supplementary material for: Iron Insufficiency Compromises Motor Neurons and Their Mitochondrial Function in Irp2-Null Mice
Source: PLoS One. 2011 Oct 7;6(10):e25404. doi: 10.1371/journal.pone.0025404 (PMC3189198; doi:10.1371/journal.pone.0025404)
Supplement: Table S1 — Summary of two-way ANOVA pairwise multiple comparisons (Tukey's Test), computed to evaluate the effect of age and genotype on the number of Myelin Dense Bodies (MDBs) in ventral spinal cord sections (average values are summarized in Figure 1C ). Two factors (age, genotype) were used for comparison and p<0.05 was used for statistical significance. (PDF) [file pone.0025404.s006.pdf]

Supplemental Table S1

Comparison	Difference of means	p	$p < 0.05$
Comparisons for factor; **genotype within 4 months**			
Wt vs *Irp1*$^{+/+}$;*Irp2*$^{-/-}$	22.75	<0.001	Yes
Wt vs *Irp1*$^{+/-}$;*Irp2*$^{-/-}$	49.75	<0.001	Yes
Irp1$^{+/+}$;*Irp2*$^{-/-}$ vs *Irp1*$^{+/-}$;*Irp2*$^{-/-}$	27.00	<0.001	Yes
Comparisons for factor; **genotype within 7.5 months**			
Wt vs *Irp1*$^{+/+}$;*Irp2*$^{-/-}$	42.25	<0.001	Yes
Wt vs *Irp1*$^{+/-}$;*Irp2*$^{-/-}$	86.25	<0.001	Yes
Irp1$^{+/+}$;*Irp2*$^{-/-}$ vs *Irp1*$^{+/-}$;*Irp2*$^{-/-}$	44.00	<0.001	Yes
Comparisons for factor; **genotype within 12 months**			
Wt vs *Irp1*$^{+/+}$;*Irp2*$^{-/-}$	52.31	<0.001	Yes
Wt vs *Irp1*$^{+/-}$;*Irp2*$^{-/-}$	158.54	<0.001	Yes
Irp1$^{+/+}$;*Irp2*$^{-/-}$ vs *Irp1*$^{+/-}$;*Irp2*$^{-/-}$	102.22	<0.001	Yes
Comparisons for factor; **age within Wt mice**			
4m vs 7.5m	0.25	0.999	No
4m vs 12m	0.03	0.999	No
7.5m vs 12m	0.22	1.000	No
Comparisons for factor; **age within *Irp1*$^{+/+}$;*Irp2*$^{-/-}$ mice**			
4m vs 7.5m	19.75	0.003	Yes
4m vs 12m	29.60	<0.001	Yes
7.5m vs 12m	9.842	0.177	No
Comparisons for factor; **age within *Irp1*$^{+/-}$;*Irp2*$^{-/-}$ mice**			
4m vs 7.5m	36.75	<0.001	Yes
4m vs 12m	108.82	<0.001	Yes
7.5m vs 12m	62.75	<0.001	Yes
